# Combining rules and dialogue: exploring stakeholder perspectives on preventing sexual boundary violations in mental health and disability care organizations

**DOI:** 10.1186/s12910-022-00786-9

**Published:** 2022-05-03

**Authors:** Charlotte Kröger, Eva van Baarle, Guy Widdershoven, Roland Bal, Jan-Willem Weenink

**Affiliations:** 1 Department of Ethics, Law and Humanities, Amsterdam UMC location Vrije Universiteit Amsterdam, Amsterdam, The Netherlands; 2 Netherlands Defense Academy, Breda, The Netherlands; 3 Erasmus School of Health Policy & Management, Erasmus University Rotterdam, Rotterdam, The Netherlands; 4 Department of Ethics, Law and Humanities, Amsterdam Public Health Research Institute, Amsterdam UMC location Vrije Universiteit Amsterdam, De Boelelaan 1089a, Amsterdam, The Netherlands

**Keywords:** Sexual boundary violations, Sexuality, Mental healthcare, Disability care, Healthcare organizations

## Abstract

**Background:**

Sexual boundary violations (SBV) in healthcare are harmful and exploitative sexual transgressions in the professional–client relationship. Persons with mental health issues or intellectual disabilities, especially those living in residential settings, are especially vulnerable to SBV because they often receive long-term intimate care. Promoting good sexual health and preventing SBV in these care contexts is a moral and practical challenge for healthcare organizations.

**Methods:**

We carried out a qualitative interview study with 16 Dutch policy advisors, regulators, healthcare professionals and other relevant experts to explore their perspectives on preventing SBV in mental health and disability care organizations. We used inductive thematic analysis to interpret our data.

**Results:**

We found three main themes on how healthcare organizations can prevent SBV in mental health and disability care: (1) setting rules and regulations, (2) engaging in dialogue about sexuality, and (3) addressing systemic and organizational dimensions.

**Conclusion:**

Our findings suggest that preventing SBV in mental health and disability care organizations necessitates setting suitable rules and regulations and facilitating dialogue about positive aspects of sexuality and intimacy, as well as about boundaries, and inappropriate behaviors or feelings. Combining both further requires organizational policies and practices that promote transparency and reflection, and focus on creating a safe environment. Our findings will help prevent SBV and promote sexual health in mental health and disability care organizations.

## Introduction

The World Health Organization stresses that good sexual health requires “a positive and respectful approach to sexuality and sexual relationships, as well as the possibility of having pleasurable and safe sexual experiences, free of coercion, discrimination and violence” [1]). Unfortunately, sexual health is sometimes negatively affected by sexual boundary violations (SBV) in healthcare settings. There are different definitions for SBV [2, 3]. We define SBV as any harmful and exploitative sexual transgression that occurs in the professional–client relationship [see for instance 4], where the “client” is any person receiving mental health or disability care. SBV can be clear-cut cases of sexual abuse, but may also be more subtle boundary violations such as unnecessarily inquiring about a client’s sexual history, physical touching, unwarranted comments, and inappropriate jokes [5, 6]. SBV are serious ethical infractions that go against professional imperatives and can have severe effects on clients, such as self-blame, depression, post-traumatic stress, and difficulty engaging in future sexual relationships [7–10].

There are inherent power disparities between clients and healthcare professionals. Clients are dependent on those that care for them. Their vulnerable position in the care relationship means that any sexual conduct between a healthcare professional and their client cannot be consensual. Nonetheless, power gaps are sometimes misunderstood and vulnerabilities can be misinterpreted as voluntary behavior between consenting adults [11, 12]. Boundaries generally define the expected and accepted psychological and social distance between professionals and clients, and have the purpose to keep both safe and secure in their roles and identities [13, 14]. However, personal perceptions of boundaries may differ and it is not always easy to determine when a boundary is being violated [5]. The road to boundary violations is often described as a slippery slope, during which a professional’s neutrality is slowly eroded [15, 16].

The actual prevalence of sexual contact between clients and professionals is probably underestimated. Self-reported data have shown that more male professionals have had intimate contact with clients (7.1–10.1%) than female professionals have (1.9–3.5%) [2]. Dutch disciplinary tribunals have handled 90 disciplinary complaints related to sexual behavior between licensed healthcare professionals and clients between 2015 and 2020, half of which originated from mental healthcare [17]. However, legal cases and self-reported data are unlikely to reflect actual prevalence [18]. Indeed, the proportion of professionals knowing a colleague who has been sexually involved with a client (38–52%) or whose clients reported being involved with a previous therapist (22–26%) is notably higher [2]. Additionally, a recent cross-sectional observational study of 2503 clients in Germany showed that 56% of female and 17% of male participants reported sexual contact with or harassment by a healthcare professional [11] and other studies have argued that sexual misdemeanor and sexual abuse are frequently underreported in healthcare [14, 19, 20]. Although the exact prevalence of SBV remains unknown, it is clear that a significant proportion of professionals and clients are confronted with SBV in healthcare. SBV in healthcare has often been considered a psychological problem of individual professionals rather than a group or organizational problem [21]. Violations are usually managed by targeting the guilty professional, for example through disciplinary measures or mandated educational courses on sexual boundaries [22–26]. Furthermore, measures to prevent SBV tend to emphasize the legal and ethical conduct of the individual professional, even though appealing to the professional ethics of potential perpetrators may be ineffective [27]. While the role of the individual is important, healthcare organizations have a responsibility to develop and implement policies and practices on a systemic level that prevent SBV and facilitate good sexual health. Still, existing studies on SBV are mainly small-scale and restricted to the perceptions of a specific healthcare organization [28, 29]. More insight is needed about (experiences with) overarching organizational mechanisms and strategies that can contribute to or may potentially hinder the prevention of SBV in healthcare practice.

Preventing SBV, particularly between clients and professionals, is a moral and legal imperative for healthcare organizations and systems. Literature on preventing SBV often concerns the importance of establishing codes of conduct, formulating organizational guidelines or generally argues for more training and education. Although these elements are highly important and necessary, SBV occur to this day and empirical insight on the actual experiences with these preventive approaches in practice are lacking. We explore experiences and perspectives of policy advisors, regulators, healthcare professionals and other relevant experts in order to gain insight into how SBV can be prevented on organizational, management, team and individual care-relationship levels within mental healthcare and disability care organizations in the Netherlands. We interviewed stakeholders with personal and professional expertise with SBV in these settings, including policy advisors from various national associations, healthcare regulators, healthcare professionals and other experts.

## Methods

### Setting

SBV occur in various healthcare contexts, particularly in residential settings for vulnerable groups, where professionals and clients have long-term relationships with numerous private and personal encounters [2]. In this study, we focus on two such risk settings: mental healthcare facilities (inpatient, residential, and outpatient) and care settings for adults with mild to moderate intellectual disabilities. While acknowledging the inherent differences in client populations and care practices between and within these care contexts, both are characterized by long-term and intimate care relationships and increased (layers of) vulnerabilities. For instance, in the Netherlands, 72% of women and 44% of men with an intellectual disability have experienced forms of sexual abuse, either within or outside their care environment in 2011, with some of the perpetrators being professionals [30].

We further focus on disability and mental healthcare because the Dutch Health and Youth Inspectorate identified these sectors as specific risk environments for SBV and has called for more research on the prevention of SBV in both contexts. This study is part of a larger research study that is financed by an independent, Dutch governmental funding agency (ZonMw) that responded to the need articulated by the Inspectorate to explore overarching perspectives on preventing SBV in mental health and disability care. While challenges around sexuality and SBV can be different between the two settings, we are interested in detecting possible commonalities and preventive strategies that may be relevant to both.

### Study design

Between March and December 2020, we carried out explorative, qualitative interviews with policy advisors, healthcare regulators, healthcare professionals and other experts involved in the policy and practice of addressing SBV in mental healthcare and disability care in the Netherlands. We chose these stakeholders because their significant expertise enabled them to provide a comprehensive perspective on the subject. We conducted 13 semi-structured interviews with 16 participants to obtain insight into their personal and professional experiences with SBV and their perspectives on how SBV may be prevented in mental healthcare and disability care organizations in the Netherlands. Three interviews were conducted with two participants simultaneously. Our interviews were guided by a topic list. We used inductive thematic analysis to interpret our data [31].

### Participant selection and sampling

To obtain a broad spectrum of perspectives, participants were recruited by purposive sampling and subsequently through the snowball method. All 16 participants were selected because they had professional, and sometimes also personal, experience with addressing and dealing with instances of SBV in policy and practice within mental healthcare or disability care.

The researchers first identified national associations for healthcare professionals from relevant fields including, for instance, the national association for disability care. We either sent a general email to the secretary of these associations, inquiring for interview partners with relevant expertise in the mental health and/or disability care sector, or contacted policy advisors directly if their contact information and area of expertise was available online and matched this study. Six participants were policy advisors. One worked for a large long-term care organization and one was also a nurse. Their expertise ranged from developing policies on patient safety, restraint or preventing boundary violations to enabling positive experiences of sexuality and facilitating good quality of care.

We also recruited healthcare professionals and regulators from the professional network of the authors and via our initial participants. Five participants were healthcare regulators working in mental healthcare, disability care, or the investigations and fines office of the Dutch Health and Youth Inspectorate. All had handled SBV and sexual assault cases. One participant was a doctor and one was a psychologist, both of whom were also sexologists working in disability care. One participant was a lawyer in mental healthcare, one was a former police officer specialized in sexual assault cases, and one was a peer-expert and chair of a client advisory board for persons with intellectual disabilities. Two participants had personally experienced severe SBV in residential healthcare contexts as former clients. Thirteen participants were female and three were male. To ensure confidentiality, we categorized the participants into four groups: policy advisors, healthcare regulators, healthcare professionals, and other ‘experts’, the latter referring to the peer-expert, lawyer or former police officer.

### Data collection

The first, second, and last author conducted 12 video interviews using Zoom and one interview by phone. Interviews lasted approximately 60 min and were guided by a topic list. Topics included questions about the participants’ (l) personal and professional experiences with and views on SBV, (2) perspectives on the role of their organization in addressing SBV in policy and practice, and (3) views on good and bad practices regarding the prevention of SBV among healthcare professionals and within mental health or disability care organizations. The researchers added topics to the topic list when new themes were generated. The interviews were conducted until data saturation was reached, i.e., when no novel themes were produced after participant interviews [32]. All interviews were audio recorded and transcribed verbatim.

### Data analysis

Instead of using an existing framework of analysis, we used inductive thematic analysis to interpret our data [31]. This allowed us to explore and identify common themes and patterns regarding participants’ practical experiences with and perspectives on how SBV can be prevented on organizational, management, team, and individual care-relationship levels within and between mental healthcare and disability care in the Netherlands. To minimize researcher biases, we analyzed our data in an iterative process. The first, second, and last author independently read the transcripts to familiarize themselves with the data. The first and second authors then coded the transcripts individually using inductive coding techniques. To increase inter-rater reliability, the first, second, and last author compared emerging themes and discussed inconsistencies at different stages of the data analysis process. We also discussed our own role and perspectives as researchers in these meetings to minimize analytical bias. The third and last author provided feedback on the rigorousness of the themes. Emerging patterns of meaning were discussed on multiple occasions among all authors until intersubjective agreement was reached on the central themes.

### Research ethics

This study was conducted in accordance with quality criteria and research ethics. To ensure confidentiality, the interview data were anonymized and coded during handling, transport, and storing. All participants provided oral informed consent to participate in the study. The Medical Ethical Review Committee of the Amsterdam UMC, location VUmc, confirmed that the Dutch Medical Research Involving Humans Act (WMO) did not apply. Additional approval was not required.

## Results

We found three main themes regarding stakeholders’ perspectives on preventing SBV in mental health and disability care organizations: (1) setting rules and regulations, (2) engaging in dialogue about sexuality, and (3) addressing systemic and organizational dimensions. All participants acknowledged that developing and implementing strategies to prevent SBV is a complex endeavor, in which different approaches may strengthen or conflict with each other.

### Setting rules and regulations

The first theme concerned setting rules and regulations to prevent SBV in mental health and disability care organizations. Participants acknowledged that clear guidelines are necessary to clarify what professional and organizational standards consider (in)appropriate sexual behavior to be, what constitutes professional integrity and how managers and professionals ought to act and respond to inappropriate behavior. However, most participants also said that prohibitive rules alone may create taboos and may be insufficient to prevent SBV. When referring to ‘rules’ or ‘regulations’ in this article, we mean both national guidelines and locally developed ward or organization-based rules that can differ across the country and within organizations.

“During a care relationship, it’s not allowed, it’s never allowed. That is a very clear statement from the inspectorate. And I think that we can all endorse this.” (Expert)

Also, there was the recognition that rules can support healthcare professionals, particularly when talking about sexuality and (presumed) boundary violations with clients.

“Some professionals search for roadmaps and rules, they want handholds and certainty […].” (Healthcare professional)

However, participants also argued that defining boundary violations, determining when they occur and developing appropriate organizational policies can be challenging and complex. This complexity, for instance, concerns putting a hand on a shoulder of a client who is crying or forbidding all adult clients who are involved in a romantic relationship to sleep in a room together, out of fear for boundary violations to occur. Most argued that this complexity cannot always be fully captured by guidelines alone and that guidelines may oversimplify SBV.

“And then there are some rules that you think, huh … [is this really] useful? … The question is what is boundary violating and for whom. … This is about dialogue. … I can imagine that professionals are very happy with this [guidelines], like: ‘Oh, then I know what I have to do’. … Meanwhile I think: it’s not that simple. … And you see this a lot of course, that there are all these guidelines, protocols, step-by-step plans … but, in practice, it’s not that simple.” (Healthcare professional)

Participants also acknowledged that rules alone are insufficient for preventing SBV and that rules may even wrongly suggest that SBV can always be prevented, controlled and managed by formulating appropriate guidelines only.

“But this [legislation on SBV] is the illusion of power. It is the illusion, in my view, that you can control and board up everything and thereby prevent that sometimes things can happen that people don’t want. Either in that moment, or later. . . It’s the illusion that you can control something.” (Expert)

Participants also indicated that rules may lead organizations to deal with issues of sexuality and intimacy in an inflexible and limited way. They argued that setting rules alone may not be sufficient to prevent SBV because rules can oversimplify the reality and complexity of SBV and create an environment in which all relationships and interactions are so controlled that even talking about sexuality, intimacy, or boundaries becomes taboo.

“So we tried to help the organizations formulate policies [to prevent SBV]. What is often an issue in organizations is that their approach is very black and white. There was an organization that even prohibited any kind of relationship. Also between employees. Men and women were even separated between departments. A rather desperate attempt which creates an even bigger taboo.” (Policy advisor)

In summary, prohibitive rules and regulations were perceived as necessary for preventing SBV between professionals and clients, particularly for preventing sexual abuse. Guidelines were also described as important for defining professional conduct, as they offer clarity and support to professionals in preventing SBV. However, focusing on rules alone to prevent SBV is likely oversimplistic, and insufficient to inhibit *all* inappropriate behaviors. It may even make sexuality and intimacy a taboo, rather than prevent unwarranted sexual behaviors and boundary violations.

### Engaging in dialogue about sexuality

In our second theme, participants indicated that engaging in open dialogue about sexuality and intimacy in healthcare organizations is an important prerequisite for defining and preventing boundary violations between and among professionals and clients.

“I think that it is particularly important to engage in conversations with professionals, colleagues, and management. Like … how do you really pay attention to the subject of sexuality? How do you talk about it … and also the negative aspects of it? Paying attention to sexuality, the positive aspects, contributes to the prevention of sexual abuse.” (Policy advisor)

We also identified two subthemes within this theme. The first subtheme was that sexuality and intimacy need to be discussed openly with clients—particularly those with intellectual disabilities—to promote sexual health. The rationale behind this is that open conversations can help clients define and understand their own and others’ boundaries and understand what is healthy sexual behavior and what is not. The second subtheme was that professionals should talk about their own and their clients’ sexuality within teams, as sexuality is an inherent aspect of being human, also in professional settings.

### Discussing sexuality with clients

Participants said that discussing sexuality with clients remains taboo. Many argued that healthcare professionals find talking about sexuality or intimacy uncomfortable and challenging because it is a sensitive and personal subject. However, most participants also argued that breaking this taboo and engaging in open conversations is important for clients’ sexual health and may help them to define and acknowledge their own and others’ sexual boundaries and engage in non-violating behaviors. This theme was particularly recognized in the context of intellectual disability care.

“I think that it used to be a taboo subject for all healthcare institutions. In all treatment plans and care plans it said, about sexuality: ‘not applicable’.” (Healthcare professional)

“People with a disability also have a sexual life, independently of their intellectual ability. They can develop fully physically and definitely also have sexual needs. So that is definitely an important starting point for thinking about sexuality and sexual abuse.” (Policy advisor)

“[To] talk about the subject [sexuality] … is also important for clients, that they also receive information about sexuality. Then they can also define their own boundaries much better.” (Healthcare professional)

Participants also indicated that talking about infatuations of clients towards professionals, rather than ignoring them, may prevent unwarranted accusations of SBV towards professionals in mental health and disability care.

“You can see this in infatuations from professionals to clients. But this also happens the other way around of course. That clients are infatuated with professionals. And yes, I have really learned this during my studies in sexology. That, well, if there is no room for infatuation, to talk about it, then this can sometimes result in an unwarranted accusation.” (Healthcare professional)

Further, all participants noted that, especially in disability care, sexual boundaries of clients might also be violated by other clients. Perceptions of sexual boundaries differ, which makes it difficult to define what is consensual and what is a boundary violation.

“I also notice that it [sexual intercourse] is sometimes easily interpreted as a boundary violation. For instance, when two clients with a disability are ‘caught in the act’ this is often seen as, well, a boundary violation. But, this might have been consensual, right?” (Healthcare professional)

Participants argued that healthcare professionals may find it difficult to address the sexuality of their clients for different reasons, including being uncomfortable with the topic of sexuality itself, the characteristics of a particular client, or conflicting perspectives on what is considered sexually appropriate for a client.

“Because a client can have the intellectual abilities of a 2-year-old, but the sexual development still progresses … healthcare professionals find it difficult to guide clients in this subject.” (Regulator)

Many participants also said that conversations about intimacy and sexual needs can be complicated further when parents or legal guardians of clients with intellectual disabilities are involved.

“You have the experience of the care professionals, the care providers that say that it [sexuality] is part of the development of the client, but the client has parents that say that their son or daughter isn’t there yet. So, to start the conversation, that is very difficult.” (Regulator)

Despite stressing the importance of addressing sexuality, sexual needs, and sexual boundaries, participants also said that nobody should be forced to talk about sexual experiences, desires, or needs. A professional’s personal experiences can define their own boundaries and impact their openness and willingness to talk about others’ sexuality.

“It can be good [to talk about sexual experiences], but it can also be difficult for the professional. It depends what the person has experienced, if it is easy to communicate with each other … because you also don’t know what the professional has experienced with sex, bad or good things … if the client or professional are both open for talking about it.” (Expert)

In summary of the first subtheme, participants argued that acknowledging the existence of sexuality and intimacy in healthcare is important for preventing SBV and false accusations of SBV. Further, discussing sexuality with clients, particularly those with an intellectual disability, is essential to helping them define and defend their personal boundaries, even if these conversations are challenging and may be perceived as uncomfortable.

### Open conversations about sexuality within teams

Openly discussing sexuality and potential risks for SBV within teams was considered important for preventing SBV in mental health and disability care. Participants argued that having regular open, dialogical conversations about sexuality within teams (for example about feeling attracted to a client) is needed to break existing taboos on “unprofessional” behavior and to create more understanding of sexual boundaries. Participants said that discussing sexuality and feelings of intimacy helps professionals to define appropriate behaviors and prevent inappropriate ones, such as acting on feelings or transgressing clients’ boundaries.

“Talk about it, just talk about it with each other. You should actually, just like, when guiding clients, address it as a topic. So also among each other, among professionals, and how they deal with clients. Just let it be an open topic…” (Healthcare professional)

“Because it [sexuality] is a lifelong theme. You won’t get there by letting someone participate in a one-time online training. Or by discussing it once in a team meeting. It is something that has to be discussed many times.” (Policy advisor)

Participants also argued that talking about emotional attraction and accepting that sexual desires and feelings can arise can prevent professionals from acting on them. Although normalizing such feelings may be uncomfortable, especially in a professional setting, participants considered this an important factor in preventing SBV and helping professionals to recognize sexual boundaries better.

“It’s just good to talk about it…. Of course, it is uncomfortable. I have different feelings than I am allowed to have professionally. To normalize these feelings. That is what interventions, especially preventive interventions, should focus on. Also, within a healthcare team, so much intimacy is shared: about patients, life, and death. Especially in mental healthcare. That is so intimate that you start to wonder: how can it be that you do not discuss your own sexual feelings towards clients?” (Policy advisor)

“And yes, we see a lot of the last category. Relationships between healthcare professionals and a client. This can be a very intimate relationship, where they even marry or have children . . . it is really about awareness within this sector [mental healthcare]. Everybody knows the professional norms and understands that it [SBV] is not okay, but it still happens. It overcomes them. We hear this a lot in conversations with healthcare professionals. But you really should be at the forefront with this. On the preventive side. How safe is it to acknowledge this in your team, that you love a patient. And what you will do. And what you should do.” (Regulator)

In summary of the second subtheme, participants suggested that sexuality, intimacy, and sexual desires are omnipresent in healthcare settings. Many also said that professionals who engage in SBV (for instance by starting a romantic relationship with a client) claim to be unaware that they have crossed a professional boundary. The core message was that, to prevent SBV and generate awareness of professional boundaries and inappropriate sexual behavior, sexuality ought to be talked about within teams and among professionals. Rules, regulations and policies within organizations may play a role here by placing emphasis on facilitating open dialogue and formulating strategies on how this may be implemented in practice.

### Addressing systemic and organizational dimensions

Another theme concerned the systemic and organizational dimensions needed to promote open communication about vulnerabilities and how to prevent boundary violations. We identified two subthemes here. First, creating an organizational culture that facilitates transparency, openness about hierarchies and power relationships, and supports positive role models. Second, facilitating an environment within organizations where professionals feel safe to address SBV.

### Fostering a supportive organizational culture

Participants emphasized the importance of organizational culture in preventing and calling out SBV within teams.

“While talking about this, I think that this also depends on the culture of the organization …” (Healthcare professional)

“At some point it becomes a sort of culture … it became a culture in which these type of topics [SBV] were assuaged … or addressed with a joking undertone … And in this case it was predominately women I think, who then said, well I am laughing with them, but actually this is not okay.” (Policy advisor)

Power relationships and hierarchy were also described to play a large part in preventing or encouraging SBV. Transparency and fewer power disparities were seen as supportive organizational mechanisms to help individuals prevent SBV.

“There’s a lot that has to do with power. This is also shown in #metoo where there’s a lot of violation of power. Also this is especially difficult for people with a disability, that they always have a sort of dependency relationship with people that can exercise power over them.” (Policy advisor)

“The more transparent an organization is, the more influence and control employees have, and there are fewer hierarchical power relationships, then you will really remove a bit of the risk [of SBV].” (Expert)

Participants also argued that having role models in management can be an important factor in facilitating a more open culture in which potential SBV are signaled and discussed, also before they occur.

“And even at the beginning I think, there are a few first steps that have to be taken. Where you have a feeling, maybe a gut feeling, like: well, this is actually not okay! (…) It’s in the first small steps (…) where we, altogether, because of the group culture or something, don’t say anything… but yes, I think it’s really about daring to speak up… also in care, that you need role models at different levels of the organization, where everybody notices: oh yes, yes you can do it like that. You can just say: hey … we don’t do this here. Instead of managers who laugh with you or talk with you or do it [SBV] themselves.” (Expert)

In summary, participants maintained that the organizational culture, including transparency about hierarchies and power relationships, and positive role models—particularly in management positions—contributes to preventing SBV in mental health and disability care organizations.

### Creating a safe environment

Participants also recognized that creating a safe environment in which professionals can be vulnerable is important for open discussions about sexual boundaries and insecurities and for preventing SBV.

“I think that safety, feeling safe, is a precondition to talking about sexuality in the first place. […]. Especially that connection and the feeling that you are not alone, that you may show your insecurities.” (Healthcare professional)

Participants also said that not addressing severe cases of SBV can make the atmosphere within an entire organization feel unsafe and perpetuate further SBV.

“So all the layoffs [due to SBV] have happened, but they are not followed up on. It becomes a type of taboo. It becomes a sort of trauma for an entire hospital. The camps are divided. Yes, some say ‘oh he was such a good man’… colleagues are often left in disbelief. Yes, this was quite traumatizing… and then you get a sort of atmosphere within a team in which sexual boundary violations actually get a chance, because there is no communication anymore … people become isolated.” (Policy advisor)

Some participants proposed that employing a special task employee (or confidentiality advisor) and engaging in structured dialogues, for instance through moral case deliberations, might allow preventive conversations before SBV occur.

“What may help is if organizations organize moral case deliberations, or have a special task employee that is easily accessible and can be called whenever you encounter challenges. Somewhere where people can come with their questions, or situations that they find difficult. Where a conversation is facilitated.” (Policy advisor)

In summary, participants argued that a safe organizational environment can prevent SBV by facilitating open communication. Participants also warned that not following up on (severe) SBV can create organizational trauma that may perpetuate further boundary violating behavior. Facilitating structured dialogues and employing a special task employee were suggested as two ways to facilitate conversations about difficult situations and to prevent SBV.

## Discussion

Based on their experiences, our participants identified three complementary ways to prevent SBV in mental healthcare and disability care organizations: 1) setting rules and regulations, 2) engaging in dialogue about sexuality, and 3) addressing systemic and organizational dimensions. These strategies do not stand alone but should be combined. In this section, we reflect on these themes individually and focus on different challenges associated with preventing SBV in healthcare organizations.

1. **Setting rules and regulations**

Participants acknowledged that rules and regulations are necessary to clarify boundaries and what constitutes inappropriate sexual behavior in mental health and disability care organizations. This is supported by national guidelines and international literature, which also show that rules are necessary to prevent SBV and to protect clients. The Dutch Healthcare Inspectorate has, for instance, developed a brochure that advises healthcare organizations to develop SBV guidelines as part of a professionals’ employment contract [33]. Research into SBV has identified the psychological aspects of individual healthcare professionals that may lead to SBV [e.g., 34] and emphasis has been placed on individual responsibility to prevent SBV rather than on organizational responsibility [21].

However, our participants also argued that prohibitive rules, regulations, and policies are not enough to prevent SBV. Imposing strict rules alone, can create the illusion that guidelines are sufficient to prevent SBV and create a taboo that hinders open communication about everything that has to do with sexuality and intimacy, also among clients. Rather than eliminating SBV, such a taboo may even create a systemic blind spot and lead to SBV occurring subversively and unnoticed. In their study on sexuality among inpatients in a secure mental healthcare facility in England, Ravenhill et al. [35] discovered a similar discourse as we observed in this study on SBV between clients and healthcare professionals. The authors “identified constructions of inpatient sexuality within two overarching and conflicting discourses: one of the normalcy and legitimacy of sexual expression in human experience; and the other of risk, wherein sexuality needed to be regulated and obstructed” [35]. While the context of our study is different, our data suggests a similar dichotomy in stakeholder’s perspectives on the possible—and unintended—effects of prohibitive organizational rules and regulations on SBV between health-care professionals if these are not also accompanied by the space to address sexuality, intimacy and feelings of attraction as an aspect of human and professional life.

Our interviews suggest that organizations face the complex challenge to simultaneously develop clear, normative rules that prevent SBV while also creating an environment in which professionals can talk freely about sexuality and express their doubts and sexual attraction *before* violations occur. We argue that while clear behavioral rules and regulations are necessary, prohibitive norms alone are not sufficient to prevent all forms of SBV in mental health and disability care organizations. Therefore, we encourage healthcare organizations to develop policies that set behavioral norms, rules and guidelines on professional integrity *and* also actively encourage openness and recurring dialogue on sexuality, intimacy and boundaries, as an additional mechanism to prevent gradual violations. The road to SBV can be a slippery slope [15] that requires dialogue to occur in order to identify and discuss boundaries before they are violated.

2. **Engaging in dialogue about sexuality**

Participants also argued that dialogical practice and open communication about sexuality can prevent SBV by tackling existing taboos and promoting sexual health. Two subthemes were important here. First, sexuality and intimacy ought to be discussed openly with clients to help them live sexually healthy lives and recognize boundaries. Second, professionals should address their own sexual feelings within teams to define boundaries and prevent violations.

### Discussing sexuality with clients

Our participants argued that creating the space to openly discussing sexual desires, health, experiences, and boundaries with clients is needed to eliminate taboos about sexuality in mental health and disability care organizations. Previous research has shown that sexuality and sexual health needs of clients are inadequately addressed in mental health [36–38] and disability care settings [39]. While many healthcare professionals acknowledge the importance of addressing sexuality and intimacy as part of holistic, recovery-oriented care, they may feel unprepared, uncomfortable and not have sufficient training to deal with these issues [39, 40]. Professionals’ personal experiences with SBV may further contribute to how sexuality is approached in a care relationship [36]. Another difficulty is that clients may not always be able to set appropriate boundaries, for example if they have previously experienced sexual abuse [30, 41, 42]. Our participants said that talking to clients about sexuality is challenging but can help them to define boundaries, avoid inappropriate behaviors and promote sexual health. However, these conversations should not be forced as some clients may not want to talk about their sexual preferences or experiences. Nonetheless, creating the space for them to do so may be an important first step to preventing SBV.

Our findings show that, to promote sexual health and structurally prevent SBV on an organizational level, organizations ought to take a broad approach to SBV in policy and practice that includes fostering sensitivity to the subject of sexuality as a whole. This means training and supporting professionals to engage in dialogue on sexual needs, intimacy, and boundaries with clients.

### Open conversations about sexuality within teams

Participants also highlighted the importance of breaking the taboo on sexuality and discussing sexual feelings and desires with other healthcare professionals. They maintained that openly talking about sexuality and risk perceptions within teams is important to create awareness, define boundaries, and prevent violations while also removing some of the taboo on sexuality.

Normalizing conversations among professionals about sexuality and acknowledging inappropriate sexual feelings may prevent boundary violations, yet these conversations are challenging. A high prevalence of sexual and romantic attraction between professionals and clients has been reported, particularly in mental healthcare [2, 34, 43]. Talking about these sexual feelings and desires can help professionals to recognize and handle these feelings, but sexual desire and attraction to clients remains a taboo subject [44]. Vesentini et al. (2021) recently identified several reasons why professionals may not want to talk about these issues (including different perspectives on what is professional or ethically appropriate behavior, feeling unsafe out of fear of being condemned or judged by others, or discomfort disclosing personal emotions) and suggested that being more open about sexual feelings would improve relationships between psychotherapists and clients [43]. While acting on these feelings and desires is unethical and goes against legal standards and professional integrity, our interviews suggest that disclosing sexual feelings or attraction within teams may help to define boundaries and refrain from violations, thereby contributing to the prevention of SBV in mental healthcare and disability care organizations. We argue that to normalize sexuality as an inherent aspect of human, professional and organizational life, mental health and disability care organizations ought to support healthcare professionals to structurally reflect, and facilitate dialogues on personal experiences of sexuality, intimacy and boundaries within teams. This may create awareness, shared responsibility and support professionals to acknowledge and address their sexual attraction and to define boundaries as a preventive strategy *before* violations occur. However, talking about sexuality and engaging in dialogue as a preventive strategy does not take away that if there are doubts about a professional’s integrity and interaction with a client in the short or long term, it is important for organizations to act and invoke professional codes of conduct for the safety of the client and to prevent a professional from doing harm.

3. **Addressing systemic and organizational dimensions**

Our participants identified several systemic and organizational dimensions that may help prevent SBV. We categorized these into two subthemes: creating a supportive organizational culture and creating a safe environment.

### Creating a supportive organizational culture

Our participants argued that organizational culture can affect SBV in speech and other behaviors in mental health and disability care organizations. Organizational culture reflects the vision, values, norms, and behaviors of an organization [45]. It provides a framework for what is acceptable and what is not. In the interviews, participants connected organizational culture to power relationships. Power differentials, social inequalities, and hierarchies between healthcare professionals and clients make it difficult to report or address concerns about SBV [29]. In our interviews, participants associated fewer hierarchical power relationships with greater openness and transparency and emphasized that this helped to prevent SBV.

In healthcare organizations that are, by definition and often by necessity, hierarchical, overcoming power disparities to create an inclusive organizational culture where vulnerabilities can be shared to prevent SBV is challenging. Despite the importance of organizational culture in healthcare, strategies on how to change and reform organizational culture are lacking [46]. Cultural change in healthcare organizations requires attention to and evaluation of a specific organizational contexts, support from leaders, and inclusive, implementable strategies and policies [47].

Based on our findings, we suggest that managers in mental health and disability care should be trained to support openness and vulnerability to create an organizational culture that can prevent SBV. Training managers to be aware of their own position of power, moral responsibility, and influence as role models, and to facilitate recurring dialogue and open communication within teams will promote transparency and allow professionals to challenge power dynamics. Further, discussing sexuality ought to be normalized at the system level and be captured in organizational policy.

### Creating a safe environment

Participants emphasized that a safe organizational environment is needed for professionals to share their insecurities and sexual feelings and thereby prevent them from acting on these feelings and crossing boundaries. Previous work has shown that, in situations where the organizational environment was not safe, psychotherapists did not dare to reveal and discuss their sexual feelings towards patients with others [43]. Further, participants also mentioned that not addressing cases of SBV that did already occur within organizations can lead to isolation and long-term organizational trauma that increases the risk of future violations. This is supported by work from other researchers, who stated that SBV can cause a “phenomenon of institutional paralysis that follows the pervasive denial and sense of helplessness when transgressions are suspected or overtly revealed” [48]. In healthcare, SBV create stigma [21] and have a lasting emotional impact on colleagues because of disturbed trust relationships and conflicted feelings of loyalty [28]. Also, SBV can severely damage organizational integrity and necessitate a process of “institutional recovery” involving long-term organizational self-analysis and communication about vulnerabilities [48]. Incidences of SBV may thus further perpetuate unsafe organizational environments [see also 23].

Our research adds to these perspectives by suggesting that dialogical practice can help to create a safe environment for sharing doubts, values, sexual needs, and desires and thereby prevent future SBVs. Our participants suggested, for instance, that employing special task employees (or confidentiality advisors) to promote dialogue in a safe setting could be one organizational strategy, while acknowledging that organizational responsibility for open communication about SBV should not be given to one individual only. Dialogical practice should be facilitated regularly, systemically and among different relevant stakeholders. For example, structured methods of clinical ethics support such as moral case deliberation can foster joint reflection on moral issues among professionals to facilitate openness, understanding, and transparency and to nurture moral learning [49]. Further, a good dialogical follow-up after cases of SBV is key to managing trauma and preventing future violations.

### Directions for future research

Our study provides novel insights into how SBV can be prevented in mental health and disability care organizations, but was exploratory in nature. Our findings call for further investigation of specific preventive and structural (dialogical) strategies that mental health and disability care organizations can employ to prevent SBV at different organizational levels. First, SBV remains a challenging and complex concept. It is not always easy to determine what is a boundary violation and what is not. To formulate preventive organizational strategies that respond to actual behaviors, the different forms and degrees of SBV between different stakeholders (among clients, between clients and professionals or among professionals for instance) need to be clarified within organizations. Second, observational data are needed to understand how the themes we identified apply to practical contexts. This also concerns gaining insight into how professionals and organizations can take a holistic approach to dealing with the complexity of preventing SBV on the one hand, while also facilitating positive aspects of sexuality and intimacy among patients in practice, on the other hand. Third, our participant sample is relatively small and focusses on policy advisors, regulators and healthcare professionals mostly. We recommend future research to include more (former) clients from disability or mental health care as participants, advisors or co-researchers, to examine whether their perspectives on sexuality, intimacy and SBV are in line with those discussed in this article. Fourth, combining setting behavioral rules and facilitating dialogical requires attention for specific organizational and systemic dimensions in different healthcare settings. This includes critically evaluating the effect of structurally implementing prohibitive policies and dialogical approaches, especially in contexts where hierarchies and power relationships exist.

## Conclusion

This article provides insight into stakeholder perspectives on preventing SBV in mental health and disability care organizations in the Netherlands. Preventing SBV entails a combination of establishing necessary rules and facilitating open dialogue, while acknowledging relevant organizational dimensions. Rules and regulations are necessary to provide guidance on professional conduct and integrity and to prevent harm. Open dialogue, in addition to rules, can contribute to recognizing boundaries and taking action before violations occur among or between clients and healthcare professionals.

We argue that mental health and disability care organizations have a moral responsibility to set and enact rules and to facilitate dialogue on sexuality in policy and practice: by fostering joint reflection, evaluation and moral learning on rules and regulations on the one hand, and on sexuality, intimacy and sexual feelings of both clients and professionals, on the other hand. This requires transparency about power relations and training healthcare professionals and managers to recognize, reflect on and talk about sexual feelings, needs and professional conduct imperatives before boundaries are violated, also within teams. Structurally promoting open dialogue between healthcare professionals and clients about sexuality, intimacy, and boundaries requires an open organizational culture and safe environment for dialogue to occur. Openly discussing sexuality and intimacy is essential for preventing SBV and a culture of silence may reinforce existing taboos.

## Data Availability

The data generated and analysed during the current study are not publicly available as individual participants might be identified from the interview transcripts. Any requests about availability of the data should be directed to the corresponding author.

## References

[1] World Health Organization. Sexual and reproductive health. 2006. Retrieved from https://www.euro.who.int/en/health-topics/Life-stages/sexual-and-reproductive-health/news/news/2011/06/sexual-health-throughout-life/definition

[2] Halter M, Brown H, Stone J. Sexual boundary violations by health professionals—an overview of the published empirical literature. 2007. Retrieved from https://www.professionalstandards.org.uk/docs/default-source/publications/research-paper/sexual-boundary-violations-2007.pdf?sfvrsn=79c47f20_6

[3] d’Oronzio JC (2015). Professional codes, public regulations, and the rebuilding of judgment following physicians’ boundary violations. AMA J Ethics.

[4] Plaut SM (2008). Sexual and nonsexual boundaries in professional relationships: principles and teaching guidelines. Sex Relatsh Ther.

[5] Gutheil TG, Brodsky A (2011). Preventing boundary violations in clinical practice.

[6] Kurpad SS, Machado T, Galgali RB, Daniel S (2012). All about elephants in rooms and dogs that do not bark in the night: boundary violations and the health professional in India. Indian J Psychiatry.

[7] Eastgate G, Van Driel ML, Lennox NG, Scheermeyer E (2011). Women with intellectual disabilities—a study of sexuality, sexual abuse and protection skills. Aust Fam Phys.

[8] Frueh BC, Knapp RG, Cusack KJ, Grubaugh AL, Sauvageot JA, Cousins VC (2005). Patients' reports of traumatic or harmful experiences within the psychiatric setting. Psychiatr Serv.

[9] Hook J, Devereux D (2018). Boundary violations in therapy: the patient's experience of harm. BJPsych Adv.

[10] Wigham S, Emerson E (2015). Trauma and life events in adults with intellectual disability. Cur Dev Disord Rep.

[11] Clemens V, Brähler E, Fegert JM (2021). #patientstoo—professional sexual misconduct by healthcare professionals towards patients: a representative study. Epidemiol Psychiatr Sci.

[12] Hook J, Devereux D (2018). Sexual boundary violations: victims, perpetrators and risk reduction. BJPsych Adv.

[13] Aravind VK, Krishnaram VD, Thasneem Z (2012). Boundary crossings and violations in clinical settings. Indian J Psychol Med.

[14] Sarkar S (2018). Boundary violation and sexual exploitation in psychiatry and psychotherapy: a review. Adv Psychiatr Treat.

[15] Galletly CA (2004). Crossing professional boundaries in medicine: the slippery slope to patient sexual exploitation. Med J Aust.

[16] Simon RI (1999). Therapist–patient sex: from boundary violations to sexual misconduct. Psychiatr Clin N Am.

[17] Gerritse FL, Duvivier RJ (2021). Disciplinary complaints concerning transgressive behaviour by healthcare professionals: an analysis of 5 years jurisprudence in the Netherlands. BMJ Open.

[18] Seto MC (1995). Sex with therapy clients: Its prevalence, potential consequences, and implications for psychology training. Can Psychol.

[19] DuBois JM, Walsh HA, Chibnall JT (2019). Sexual violation of patients by physicians: a mixed-methods, exploratory analysis of 101 cases. Sexual Abuse.

[20] Willott S, Badger W, Evans V (2020). People with an intellectual disability: under-reporting sexual violence. J Adult Prot.

[21] Dimen M (2016). Rotten apples and ambivalence: sexual boundary violations through a psychocultural lens. J Am Psychoanal Assoc.

[22] Spickard A, Swiggart WH, Manley G, Dodd D (2002). A continuing education course for physicians who cross sexual boundaries. Sex Addict Compuls.

[23] Swiggart W, Dewey C, Ghulyan M (2016). Spanning a decade of physician boundary violations: are we improving?. HEC Forum.

[24] Heiden JM (1993). Preview-prevent: a training strategy to prevent counselor-client sexual relationships. Couns Educ Superv.

[25] Robinson GE, Stewart DE (1996). A curriculum on physician–patient sexual misconduct and teacher–learner mistreatment. Part 2: teaching method. CMAJ Can Med Assoc J.

[26] White GE (2003). Medical students' learning needs about setting and maintaining social and sexual boundaries: a report. Med Educ.

[27] Pope KS (1990). Therapist-patient sex as sex abuse: six scientific, professional, and practical dilemmas in addressing victimization and rehabilitation. Prof Psychol Res Pract.

[28] McGarry J (2019). ‘Hiding in plain sight’: exploring the complexity of sexual safety within an acute mental health setting. Int J Ment Health Nurs.

[29] Melville-Wiseman J (2015). Professional sexual abuse in mental health services: capturing practitioner views of a contemporary corruption of care. Soc Work Soc Sci Rev.

[30] van Berlo W, de Haas S, van Oosten N. van Dijk L, Brants L, Tonnon S, Storms O. Beperkt weerbaar. Rutgers WPF and MOVISIE, Utrecht. 2011. Retrieved from https://www.movisie.nl/sites/movisie.nl/files/publication-attachment/Beperkt%20weerbaar%20%5BMOV-181899-0.4%5D.pdf

[31] Braun V, Clarke V (2006). Using thematic analysis in psychology. Qual Res Psychol.

[32] Saunders B, Sim J, Kingstone T, Baker S, Waterfield J, Bartlam B, Burroughs H, Jinks C (2018). Saturation in qualitative research: exploring its conceptualization and operationalization. Qual Quant.

[33] Inspectie voor Gezondheidszorg en Jeugd (IGJ). Het mag niet, het mag nooit. Seksueel grensoverschrijdend gedrag in de gezondheidszorg. 2016. Retrieved from https://vbag.nl/uploads/userfiles/file/Brochure%20IGZ-1.pdf

[34] Dickeson E, Roberts R, Smout MF (2020). Predicting boundary violation propensity among mental health professionals. Clin Psychol Psychother.

[35] Ravenhill JP, Poole J, Brown SD, Reavey P (2020). Sexuality, risk, and organisational misbehaviour in a secure mental healthcare facility in England. Cult Health Sex.

[36] Janssens K, Kuyper A, Van Oosten N. Kennisbundel Seksualiteit en preventie seksueel misbruik bij mensen met een beperking. 2013. Retrieved from https://www.kennispleingehandicaptensector.nl/docs/KNP/Verbeterprogramma/Seksualiteit/Kennisbundel%20-%20seksualiteit_1005.pdf

[37] Urry KL. Sexuality and sexual health in mental health care settings: perceptions of psychologists, psychiatrists, and mental health nurses in Australia (Doctoral dissertation). The University of Adelaide. 2020.

[38] Quinn C, Platania-Phung C, Bale C, Happell B, Hughes E (2018). Understanding the current sexual health service provision for mental health consumers by nurses in mental health settings: fndings from a survey in Australia and England. Int J Ment Health Nurs.

[39] McGrath M, Low MA, Power E, McCluskey A, Lever S (2021). Addressing sexuality among people living with chronic disease and disability: a systematic mixed methods review of knowledge, attitudes, and practices of health care professionals. Arch Phys Med Rehabil.

[40] Tennille J, Bohrman C, Barrenger S (2021). Behavioral health provider attitudes and beliefs about sexuality and intimacy: findings from a mixed method design. Community Ment Health J.

[41] Byrne G (2018). Prevalence and psychological sequelae of sexual abuse among individuals with an intellectual disability: a review of the recent literature. J Intellect Disabil.

[42] Stoffelen JMT, Schaafsma D, Kok G, Curfs LMG (2019). Views on sex using the nominal group technique to explore sexuality and physical intimacy in individuals with *Intellectual Disabilities*. Sex Disabil.

[43] Vesentini L, Van Puyenbroeck H, De Wachter D, Matthys F, Bilsen J (2021). Sexual feelings toward clients in the psychotherapeutic relationship: the taboo revealed. Qual Health Res.

[44] Barnett JE (2014). Sexual feelings and behaviors in the psychotherapy relationship: an ethics perspective. J Clin Psychol.

[45] Nightingale A (2018). Developing the organisational culture in a healthcare setting. Nurs Stand.

[46] Parmelli E, Flodgren G, Beyer F (2011). The effectiveness of strategies to change organisational culture to improve healthcare performance: a systematic review. Implement Sci.

[47] Johnson A, Nguyen H, Groth M, Wang K, Li Ng J (2016). Time to change: a review of organisational culture change in health care organisations. J Organ Eff: People Perform.

[48] Honig RG, Barron JW (2013). Restoring institutional integrity in the wake of sexual boundary violations: a case study. J Am Psychoanal Assoc.

[49] Weidema FC, Molewijk AC, Kamsteeg F, Widdershoven GAM (2013). Aims and harvest of moral case deliberation. Nurs Ethics.

